# Association of estimated liver fibrosis with carotid but not femoral atherosclerotic burden: the ILERVAS cohort

**DOI:** 10.3389/fendo.2025.1651689

**Published:** 2026-01-06

**Authors:** Josep León-Mengíbar, María M. Malagón, Marcelino Bermúdez-López, José Manuel Valdivielso, Reinald Pamplona, Gerard Torres, Dídac Mauricio, Eva Castro, Elvira Fernández, Assumpta Caixàs, Marta Hernández, Carolina Lopez-Cano, Ana Gordon, Rocio Guzman-Ruiz, Kenneth Cusi, Albert Lecube

**Affiliations:** 1 Endocrinology and Nutrition Department, University Hospital Arnau de Vilanova de Lleida, Lleida, Spain; 2 Obesity, Diabetes and Metabolism (ODIM) Research Group, Lleida Biomedicine Research Institute (IRBLleida), University of Lleida, Lleida, Spain; 3 Department of Cell Biology, Physiology, and Immunology, Maimonides Institute for Biomedical Research of Córdoba (IMIBIC), University of Córdoba (UCO), Biomedical Research Network center on the Pathophysiology of Obesity and Nutrition (CIBERobn), Instituto de Salud Carlos III, Córdoba, Spain; 4 Vascular and Renal Translational Research Group, Lleida Biomedicine Research Institute (IRBLleida), Renal Research Network (RICORS2040, ISCIII), Lleida, Spain; 5 Department of Experimental Medicine , University of Lleida, Lleida, Spain; 6 Department of Experimental Medicine, University of Lleida, Lleida Biomedicine Research Institute (IRBLleida), Lleida, Spain; 7 Translational Research in Respiratory Medicine, University Hospital Arnau de Vilanova and Santa Maria, Lleida Biomedicine Research Institute (IRBLleida), Biomedical Research Network Center of Respiratory Diseases (CIBERes), Institute of Health Carlos III, Lleida, Spain; 8 Department of Endocrinology and Nutrition, Hospital de la Santa Creu i Sant Pau, Biomedical Research Network Center on Diabetes and Metabolic Diseases (CIBERdem), Instituto de Salud Carlos III, Autonomous University of Barcelona, Barcelona, Spain; 9 Endocrinology and Nutrition Department, Parc Taulí Hospital Universitari, Institute for Research and Innovation Parc Taulí-Centers of Research in Catalonia (IPT-CERCA), Sabadell, Spain; 10 Medicine Department, Universitat Autònoma de Barcelona, Sabadell, Spain; 11 Division of Endocrinology, Diabetes and Metabolism, University of Florida, Gainesville, FL, United States; 12 Endocrinology and Nutrition Department, University Hospital Vall d'Hebron, Barcelona, Spain; 13 Diabetes and Metabolism Research Group, Institut de Recerca Vall d'Hebron (VHIR), CIBER of diabetes and metabolic diseases (CIBERdem), Autonomous University of Barcelona, Barcelona, Spain

**Keywords:** cardiovascular risk factors (CVRF), FIB-4 index, liver fibrosis, MASLD, metabolic dysfunction associated steatotic liver disease, NAFLD fibrosis score (NFS), subclinical atheromatous disease

## Abstract

**Introduction:**

Advanced liver fibrosis, a key complication of metabolic dysfunction-associated steatotic liver disease, has been increasingly linked to extrahepatic conditions, including type 2 diabetes, obesity, and cardiovascular disease. However, the specific association of liver fibrosis in the development and progression of subclinical atheromatous disease across vascular territories remains poorly understood. This study evaluates the utility of two non-invasive indices to predict liver fibrosis and their associations with subclinical atheromatous plaque burden and distribution.

**Methods:**

Atheromatous plaque burden (plaque presence, number, and total area) was assessed in the carotid and femoral territories via ultrasonography in 3,981 middle-aged participants without known cardiovascular disease, diabetes, or liver disease from the ILERVAS cohort (ClinicalTrials.gov Identifier: NCT03228459). The fibrosis-4 (FIB-4) and the NAFLD Fibrosis Score (NFS) were evaluated. FIB-4 risk categories were defined as low (<1.30), intermediate (1.30–2.67), and high (>2.67).

**Results:**

Participants in the intermediate and high-risk FIB-4 categories exhibited a higher prevalence of carotid atheromatous disease (56.8% vs. 49.5%, p<0.001), a greater number of plaques (p<0.001), and a larger total plaque area (p=0.007). Multivariable analyses confirmed FIB-4 as an independent predictor of carotid plaque burden (OR: 1.14, 95% CI 1.05-1.24, p=0.003), even adjusting for traditional cardiovascular risk factors. Moving from low to high FIB-4 cut-offs was associated with 12.6% higher odds of carotid atherosclerosis. NFS was also independently associated with carotid atheromatosis (OR 1.10, 95% CI 1.05–1.15, p<0.001). No significant associations were found in the femoral territory for either index.

**Conclusions:**

Estimated liver fibrosis, particularly FIB-4, is a valuable marker for identifying carotid subclinical atherosclerosis in populations without known liver disease. These findings highlight the importance of vascular territory-specific evaluations and support their utility in integrated liver and cardiovascular risk assessment strategies.

## Introduction

Liver fibrosis, a hallmark of progressive metabolic dysfunction-associated steatotic liver disease (MASLD), is emerging as a key determinant of cardiovascular (CV) risk (1). While MASLD remains the most prevalent chronic liver disease worldwide, affecting approximately 25% of the global population (2), it is the progression to liver fibrosis that seems to carry the most significant prognostic implications beyond hepatic complications (1, 3, 4). Fibrosis severity has been linked not only to advanced liver outcomes, such as cirrhosis and hepatocellular carcinoma, but also to extrahepatic conditions, including insulin resistance, type 2 diabetes mellitus (T2DM), hypertension, and atherogenic dyslipidemia (5). CV disease is recognized as one of the leading causes of mortality in patients with MASLD (6). Growing evidence suggests that liver fibrosis, along with liver dysfunction, contributes independently to this increased CV risk through systemic metabolic alterations, chronic inflammation, and endothelial dysfunction (7, 8). In addition, pro-inflammatory cytokines and oxidative stress associated with fibrosis may enhance vascular changes leading to a more severe atheromatous plaque formation (9). However, whether liver fibrosis represents an independent risk factor for subclinical atheromatosis distinct from other metabolic comorbidities such as T2DM, remains an open question (10). Similarly, it is still unclear whether assessing liver fibrosis itself, independently of a formal MASLD diagnosis, might offer an opportunity to identify individuals at increased CV risk.

Early stages of atherogenesis are characterized by the hyperplasia and expansion of adventitial vasa vasorum (VV) into the avascular intima (11). Although MASLD severity, as assessed by liver biopsy, has shown no clear association with VV expansion in individuals with severe obesity (12), the specific role of fibrosis in promoting atheromatosis has not been fully explored (13). Some studies suggest that liver fibrosis, more than steatosis itself, could correlate with increased atherosclerotic plaque burden in various vascular territories, including carotid, coronary, and femoral arteries (13, 14). In fact, it has been shown that liver fibrosis, assessed by the fibrosis-4 score (FIB-4), identifies acute coronary syndrome patients not only at higher risk of in-hospital mortality but also at increased risk of heart failure after discharge (15). In addition, longitudinal studies report that patients with steatosis are more likely to develop coronary arterial calcification (CAC) or incident carotid plaques over time (16–18). However, other studies have found no significant association between MASLD and surrogate markers of CV disease, including carotid intima-media thickness, aortic stiffness, brachial artery vasodilatory function, and CAC (8–11).

Based on the hypothesis that advanced liver fibrosis may be associated with a greater burden of subclinical atheromatous disease, we aimed to evaluate the relationship between two non-invasive liver fibrosis indices, the FIB-4 index and the NAFLD fibrosis (NFS) score, and subclinical atheromatosis (plaque presence, affected territories, number, and total plaque area) in a large population of individuals with low to moderate CV risk from the province of Lleida, Spain (19–21).

## Methods

### Study population, metabolic status, and selection of patients

The ongoing prospective ILERVAS study (ClinicalTrials.gov Identifier: NCT03228459) examines the progression of subclinical atherosclerotic disease (SAD) in individuals with low to moderate cardiovascular risk (22, 23). Of 8,330 participants recruited between January 2015 and December 2017, 3,940 had complete FIB-4 data and 3,981 NFS.

The inclusion criteria were: age 45–70 years, absence of previous cardiovascular disease (angina, myocardial infarction, stroke, peripheral artery disease, heart failure, or any vascular surgery/procedure), and the presence of at least one cardiovascular risk factor [dyslipidemia, arterial hypertension, obesity, smoking, or a first-degree relative with premature cardiovascular disease (myocardial infarction, stroke, or peripheral arterial disease] before the age of 55 years in men or 65 years in women)]. Exclusion criteria included any form of diabetes mellitus, chronic hepatitis or kidney disease, active neoplasia, hematologic disorders, life expectancy less than 18 months, or pregnancy. Regarding liver disease, participants with known chronic hepatopathies (including hepatitis B or C infection, cirrhosis, liver cancer, or other chronic liver disorders), identified through clinical history and diagnostic codes (ICD-9/ICD-10, as applicable at the time of recruitment) in electronic medical records, were excluded from the cohort. Prediabetes was defined according to the ADA criteria: fasting plasma glucose of 100–125 mg/dL or HbA1c 5.7–6.4% (24).

The prescribed treatments for hypertension (ACE inhibitors, diuretics, angiotensin receptor blockers, beta-blockers, and calcium channel blockers) and lipid management (statins, fibrates, ezetimibe, and omega-3 fatty acids) were extracted from prescription data provided by the Catalan Health Service.

### Evaluation of clinical variables

Height and body weight were measured with participants wearing light clothing without shoes. Total cholesterol levels (mg/dL) were determined from non-fasting dried capillary blood samples obtained via fingertip puncture and analyzed with the REFLOTRON^®^ Plus system (Roche Diagnostics, GmbH, Germany) (22, 23). A complete lipid profile was measured in participants with total cholesterol levels ≥ 200 mg/dL after fasting for at least 6 hours, or ≥ 250 mg/dL regardless of fasting status. Pulse pressure was calculated as the difference between systolic blood pressure (SBP) and diastolic blood pressure (DBP). Smoking status was categorized as non-smoker, current smoker, or former smoker, with the latter defined as participants who had quit smoking one year prior.

### Non-invasive methods for predicting liver fibrosis

Following a literature review to identify predictive models of liver fibrosis validated in MASLD and based on routinely available clinical data and serum markers, two indices were included in our study: the FIB-4 index and the NFS (19–21). The formulas and risk categories used to calculate these scores were: (i) FIB-4 index = (Age × AST [U/l]/(platelet count [10^9^/L] × √ALT (U/l), with the risk categories defined as <1.30 for low risk, 1.30-2.67 for intermediate risk, and >2.67 for high risk; and (ii) NFS = −1.675 + (0.037 × age) + (0.094 × BMI [kg/m²]) + (1.13 × diabetes [yes = 1, no = 0] + (0.99 × AST/ALT ratio) – (0.013 × platelet count [× 10^9^/l]) – (0.66 × albumin [g/dl]), with the risk categories defined as < -1.455 for no fibrosis, -1.455-0.675 for possible fibrosis, and >0.675 for suggestive of fibrosis.

### Assessment of atheromatous plaque burden

The bilateral carotid arteries (common, bifurcation, internal, and external) and femoral arteries (common and superficial) were examined using a Vivid-I Doppler Ultrasound system (General Electric Healthcare, Waukesha, WI, USA) equipped with a 12L-RS broadband linear probe operating at frequencies between 5 and 13 MHz. Images were acquired by trained sonographers following standardized and validated protocols (25). The sonographers were blinded to the participants’ clinical histories to avoid potential bias.

Subclinical atheromatosis was defined as the presence of any plaque in the twelve evaluated vascular regions (26). A plaque was well-defined as a focal intima-media thickness ≥ 1.5 mm projecting into the arterial lumen (27). All detected plaques were measured, and the total plaque area (cm²) was calculated (28).

### Statistical analysis

Non-normal variables were expressed as the median [interquartile range]. Categorical variables were reported as absolute frequencies. Group comparisons were performed using the Pearson’s Chi-squared test for categorical variables, whilst Mann-Whitney U test and Kruskal Wallis test were applied for quantitative variables. The association between continuous variables was evaluated using the Spearman correlation test. In line with the guideline-endorsed two-step interpretation of FIB-4 (<1.3 ‘rule out’, ≥1.3 ‘rule in’) and because only a small number of participants had high FIB-4 values (n=61), the moderate and high fibrosis categories were combined for analysis (29, 30).

A multivariable logistic regression model was developed to assess the presence of SAD, incorporating the following potential confounding factors: sex, prediabetes status, LDL cholesterol, triglycerides, pulse pressure, smoking status, and each non-invasive liver fibrosis predictor (FIB-4 or NFS) separately. The constituent components of each score were not included as separate covariates in the models to minimize multicollinearity. A multivariable lineal regression model was also performed to evaluate the number of plaques in the different territories and the total plaque area using the same covariates. Furthermore, stratified analyses were conducted by sex to explore potential sex-specific associations between FIB-4 and carotid plaque burden, applying the same set of confounding variables as in the main models. Multicollinearity between FIB-4 and covariates, including age, was assessed using the variance inflation factor (VIF), and no significant collinearity was detected (all VIF values < 5). Linearity of the association between FIB-4 and plaque was examined by fitting logistic regression models including both a quadratic term and restricted cubic splines (4 df). Model fit was assessed using likelihood ratio tests (quadratic vs. linear: LR χ²(1)=0.0004, p=0.98; spline vs. linear: LR χ²(3)=2.42, p=0.49) and the Akaike Information Criterion (AIC; 1327.4 vs. 1323.8, respectively). As no departures from linearity were detected, FIB-4 was modeled as a continuous linear predictor. Missing data were handled using a complete-case approach, whereby participants with missing values in variables included in each analysis were excluded. The proportion of missing data was low (<5% in all key variables), and therefore the potential impact on the results is expected to be minimal. All statistical analyses were conducted using STATA v.16 software, with a significant threshold set at 0.05.

### Ethical considerations

The ILERVAS study protocol received approval from the Ethics Committee of the University Hospital Arnau de Vilanova (Initial approval: CEIC-1410, 19/12/2014). All participants provided written informed consent prior to inclusion in the study. The research was conducted in accordance with the Declaration of Helsinki.

## Results

**Figure 1:**
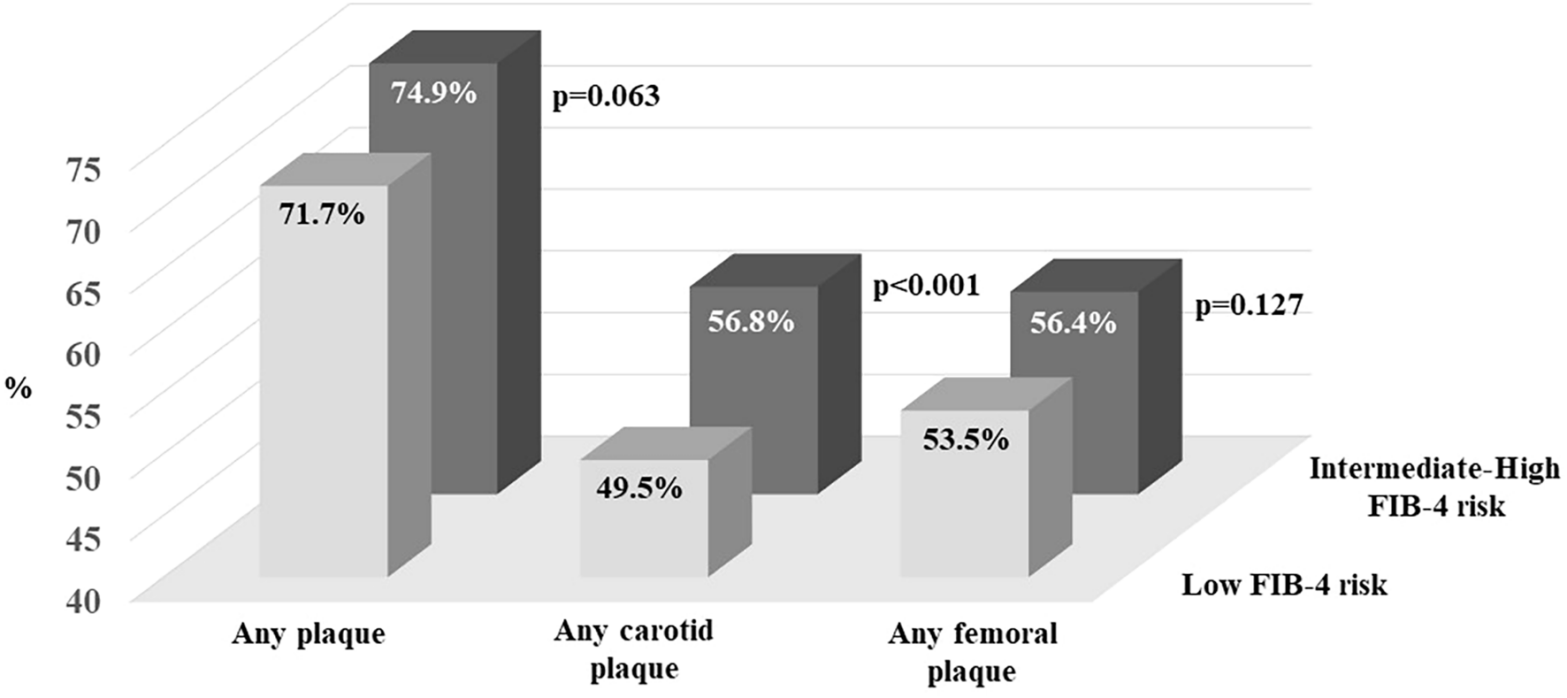
Prevalence of atheromatous disease according to the FIB-4 index thresholds. Affected territories include bilateral carotid (common, bifurcation, internal, and external) and femoral (common and superficial) arteries. FIB-4 risk categories are defined as <1.30 for low risk, 1.30-2.67 for intermediate risk, and >2.67 for high risk.

The main clinical and metabolic characteristics of the participants, stratified by FIB-4 index thresholds, are summarized in **Table 1** and **Figure 1**. According to the FIB-4 classification, most participants were categorized as low-risk for fibrosis (78.3%). As expected from the formula used to calculate FIB-4, these individuals also exhibited lower platelet counts and impaired liver function test results. In addition, participants with higher FIB-4 values showed increased pulse pressure and a greater use of antihypertensive medications. Interestingly, despite these unfavorable cardiovascular risk factors, they displayed a less atherogenic lipid profile, with no significant differences in the use of lipid-lowering medications.

**Table 1:** Main clinical characteristics, metabolic data and medical treatment in the study population according to the FIB-4 index thresholds.

Variables	FIB-4 low risk	FIB-4 indeterminate-high risk	P
N	3.085	855	–
Women, n (%)	1635 (52.9)	467 (54.6)	0.400
Age (years)	57.5 (52.5-62.5)	61.5 (55.5-65.5)	<0.001
BMI (kg/m²)	28.6 (25.8-32.0)	28.2 (25.2-31.3)	0.001
Glycaemia (mg/dl)	96 (89-104)	95 (89-103)	0.640
Platelets (10^9^/L)	254 (222-291)	207 (180-235)	<0.001
AST (U/L)	22 (19-26)	25 (21-33)	<0.001
ALT (U/L)	20 (16-27)	20 (15-31)	0.090
Total cholesterol (mg/dl)	216 (192-240)	213 (188-238)	0.053
LDL cholesterol ^a^ (mg/dl)	132 (113-154)	129 (109-155)	0.020
HDL cholesterol ^a^ (mg/dl)	55 (47-64)	56 (49-66)	<0.001
Triglycerides ^a^ (mg/dl)	119 (88-166)	114 (81-162)	0.008
Lipid-lowering agents, n (%)	600 (19.4)	186 (21.7)	0.136
SBP (mm Hg)	131 (120-142)	132 (120-144)	0.076
DBP (mm Hg)	82 (76-88)	81 (75-87)	0.028
Pulse Pressure (mm Hg)	48 (41-56)	50 (42-59)	<0.001
Antihypertensive agents, n (%)	1262 (40.9)	385 (45.0)	0.031
Current or former smoker, n (%)	1273 (41.2)	296 (34.6)	<0.001
Characteristics of atheromatous disease			
Presence of any plaque, n (%)	2214 (71.7)	641 (74.9)	0.063
Number of affected territories (n) ^b^	1.9 (0-3)	2.3 (0-3)	<0.001
Total plaque area (cm²)	0.4 (0.2-0.9)	0.6 (0.2-1.1)	0.003
Any carotid territory affected, n (%) ^b^	1530 (49.5)	486 (56.8)	<0.001
Number of carotid plaques (n)	1.0 (0-2)	1.2 (0-2)	<0.001
Carotid plaque area (cm²)	0.1 (0.1-0.3)	0.2 (0.1-0.4)	0.007
Any femoral territory affected, n (%) ^b^	1652 (53.5)	483 (56.4)	0.127
Number of femoral plaques (n)	0.9 (0-2)	1.0 (0-2)	0.086
Femoral plaque area (cm²)	0.5 (0.2-0.9)	0.5 (0.2-1.0)	0.111

Dates are expressed as median (interquartile range) or n (percentage). FIB-4 risk categories are defined as <1.30 for low risk, 1.30-2.67 for intermediate risk, and >2.67 for high risk. AST: aspartate aminotransferase; ALT: alanine aminotransferase; LDL: low density lipoprotein; HDL: high density lipoprotein; SBP: systolic blood pressure; DBP: diastolic blood pressure; BMI: body mass index. ^a^ Determination was done in cases in which total cholesterol was ≥ 200 mg/dL and after fasting for 6 h or total cholesterol ≥ 250 mg/dL regardless of fasting hours. ^b^ Affected territories include bilateral carotid (common, bifurcation, internal, and external) and femoral (common and superficial) arteries.

Regarding atherosclerotic disease, individuals with the higher FIB-4 values demonstrated a significantly greater number of affected vascular territories [2.3 (0 to 3) vs. 1.9 (0 to 3); *p* < 0.001] and a higher total plaque area [0.6 (0.2 to 1.1) cm² vs. 0.4 (0.2 to 0.9) cm²; *p* = 0.003] (**Table 1**). These differences were primarily driven by carotid artery involvement. Specifically, participants in the high-risk group exhibited an approximately 10% higher prevalence of carotid artery disease (56.8% vs. 49.5%, p <0.001), a greater number of carotid plaques [1.2 (0 to 2) vs. 1.0 (0 to 2), p <0.001], and a larger carotid plaque area (*p* = 0.007). A similar pattern of increased plaque in the carotid arteries was observed in participants with higher FIB-4 values compared to those at low risk, affecting both the right (42.8 vs 37.1%, p=0.003) and left (40.8 vs. 35.5%, p=0.005) sides. Conversely, no significant differences between groups were observed in the femoral territories.

Univariate logistic regression analysis identified FIB-4 as an independent predictor of carotid plaque presence [OR 1.30 (95% CI 1.13 to 1.49), *p* < 0.001] but not for femoral plaques (**Table 2**). Further analysis using multivariate logistic regression, confirmed that FIB-4, along with established cardiovascular risk factors such as smoking, prediabetes, elevated pulse pressure, increased LDL cholesterol and male gender, independently predicted the presence of carotid plaques [OR 1.14 (1.05 to 1.24), p =0.003] (**Table 3**). Using clinical cut-offs, moving from the low-risk threshold (1.30) to the high-risk threshold (2.67) was associated with approximately 12.6% higher odds of carotid atherosclerosis. When analyses were stratified by sex, the association between FIB-4 and the number of carotid plaques remained significant in men [OR 1.18 (1.03 to 1.36), p =0.018] but not in women [OR 1.11 (0.99 to 1.24), p =0.067]. Full results of the sex-stratified multivariable models are provided in **Supplementary Tables 1** and **2**.

**Table 2:** Univariable logistic regression model for the analysis between the presence (yes/no) of plaque and the non-invasive methods.

Variables	Odds ratio (95% confidence interval)	P
FIB-4 index		
Presence of any plaque	1.16 (0.99-1.36)	0.059
Any carotid territory affected	1.30 (1.13-1.49)	<0.001
Any femoral territory affected	1.11 (0.96-1.30)	0.149
NAFLD fibrosis score		
Presence of any plaque	1.19 (1.07-1.33)	0.002
Any carotid territory affected	1.24 (1.12-1.37)	<0.001
Any femoral territory affected	1.05 (0.95-1.17)	0.297

**Table 3:** Logistic regression model for the analysis between the presence (yes/no) of carotid plaque and FIB4-index.

Variables	Odds ratio (95% confidence interval)*	P
Smoking (yes vs. no)	1.33 (1.15 to 1.54)	<0.001
Prediabetes (yes vs. no)	1.28 (1.11 to 1.48)	0.001
FIB-4 Index	1.14 (1.05 to 1.24)	0.003
Pulse pressure (mm Hg)	1.03 (1.03 to 1.04)	<0.001
LDL cholesterol (mg/dl)	1.005 (1.003 to 1.04)	<0.001
Sex (women vs. men)	0.61 (0.53 to 0.71)	<0.001
Triglycerides (mg/dl)	1.001 (0.99 to 1.002)	0.235
Obesity (yes vs. no)	1.12 (0.96 to 1.29)	0.139
Test of fit Hosmer–Lemeshow	–	0.2837
Area under de ROC curve	–	0.6382

*Adjusted odds ratios (ORs) account for all covariates included in the regression model. LDL: low density lipoproteins. Obesity was defined as a body mass index (BMI) ≥ 30 kg/m², and prediabetes status was defined following current American Diabetes Association guidelines (fasting plasma glucose of 100–125 mg/dL or HbA1c 5.7–6.4%).

When using the NFS, participants with fibrosis-suggestive values exhibited a higher overall prevalence of atheromatous plaques (75.3% vs. 70.5%, *p* = 0.002), mainly driven by greater carotid involvement. Specifically, they showed an increased prevalence of affected carotid territories (55.4% vs. 47.9%, *p* < 0.001) and a higher number of plaques in this region (1.15 vs. 0.95, *p* < 0.001) (**Table 4**). In the unadjusted logistic regression analysis the NFS was identified as an independent predictor for the presence of any plaque, and specifically within the carotid territory (**Table 2**). In the multivariable logistic model the NFS persisted as an independent predictor for the presence of carotid [OR 1.10 (1.05 to 1.15), p <0.001] but no femoral plaque (**Table 5****).**

**Table 4:** Atheromatous disease characteristics in the study population according to the NAFLD fibrosis score thresholds.

Variables	NAFLD score no fibrosis	NAFLD score possible fibrosis	NAFLD score suggestive of fibrosis	p
N	323	1.926	1.732	–
Presence of any plaque, n (%)	228 (70.5)	1355 (70.3)	1305 (75.3)	0.002
Number of affected territories ^a^	2 (0-3)	1.97 (0-3)	2.18 (1-3)	<0.001
Total plaque area, (cm²)	0.51 (0.19-1.02)	0.46 (0.21-0.96)	0.51 (0.21-1.01)	0.306
Any carotid territory affected, n (%) ^a^	155 (47.9)	927 (48.1)	960 (55.4)	<0.001
Number of carotid plaques	0.95 (0-1)	1.01 (0-2)	1.15 (0-2)	<0.001
Carotid plaque area, (cm²)	0.18 (0.1-0.38)	0.2 (0.1-0.38)	0.21 (0.11-0.4)	0.697
Any femoral territory affected, n (%) ^a^	181 (56.0)	1013 (52.6)	966 (55.7)	0.125
Number of femoral plaques	1.06 (0-2)	0.95 (0-2)	1.03 (0-2)	0.069
Femoral plaque area, (cm²)	0.51 (0.23-0.94)	0.5 (0.24-0.94)	0.55 (0.26-0.97)	0.401

a Affected territories include bilateral carotid (common, bifurcation, internal, and external) and femoral (common and superficial) arteries.

**Table 5:** Logistic regression model for the analysis between the presence (yes/no) of carotid plaque and NAFLD fibrosis score.

Variables	Odds ratio (95% confidence interval)*	P
Smoking (yes vs. no)	1.35 (1.17 to 1.56)	<0.001
NAFLD fibrosis score	1.10 (1.05 to 1.15)	<0.001
Pulse pressure (mm Hg)	1.03 (1.03 to 1.04)	<0.001
LDL cholesterol (mg/dl)	1.005 (1.003 to 1.007)	<0.001
Sex (women vs. men)	0.62 (0.54 to 0.73)	<0.001
Triglycerides (mg/dl)	1.001 (0.99 to 1.002)	0.083
Test of fit Hosmer–Lemeshow	–	0.2045
Area under de ROC curve	–	0.6337

*Adjusted odds ratios (ORs) account for all covariates included in the regression model. LDL: low density lipoproteins.

## Discussion

Our study demonstrates that individuals in the intermediate and high-risk FIB-4 category exhibit a significantly higher overall plaque burden compared to those in the low-risk group, a finding that is largely driven by a greater prevalence of carotid, but not femoral, SAD. This finding aligns with prior research suggesting a link between MASLD and CV disease but provides a novel perspective by focusing on fibrosis-specific associations across vascular territories. Although several studies have explored the impact of hepatic steatosis, often estimated using the fatty liver index, on CV outcomes, the role of advanced liver fibrosis as a contributor to atherosclerotic disease across different vascular sites has remained largely underexplored (31–36).

Our findings highlight that liver-related fibrosis, as estimated using FIB-4 thresholds, is associated with differential patterns of atherosclerosis. Specifically, participants with intermediate and high-risk FIB-4 values showed higher prevalence, number and area of carotid plaques, while no significant associations were observed in the femoral territory. Although the observed 12.6% increased odds of carotid atherosclerosis associated with intermediate-high FIB-4 values reached statistically significance, the magnitude of this effect is moderate and should be interpreted in the context of overall clinical risk and multifactorial disease etiology. At the same time, this divergence underscores the need for tailored risk assessment strategies based on vascular site-specific mechanisms. Factors associated with chronic liver disease and leading to advanced liver fibrosis may contribute to carotid plaque formation, potentially mediated by insulin resistance, cardiometabolic risk factors linked to metabolic dysfunction and chronic subclinical inflammation (37–39). Notably, metabolic factors such as prediabetes and pulse pressure emerged as significant predictors in the multivariate model, reinforcing their role in carotid atherogenesis. Additionally, our findings raise the possibility that systemic fibro-inflammatory mediators released by the fibrotic liver may contribute to endothelial dysfunction in specific vascular territories. Experimental studies have shown that fibrotic livers release cytokines, chemokines, adhesion molecules, and extracellular vesicles, as well as lipids (mainly triglycerides), that promote systemic endothelial dysfunction (40). Moreover, the crosstalk between liver sinusoidal endothelial cells and the hepatic microenvironment is increasingly recognized as a key driver of fibrogenesis and systemic pro-inflammatory signaling (41). Whether such mechanisms preferentially affect certain vascular beds remains an open question that requires dedicated mechanistic studies.

The differential impact of fibrosis on carotid versus femoral atheromatosis may be explained by anatomical differences between these vascular territories. Femoral arteries have a distinct microscopic structure and flow dynamics compared to carotid arteries, which may make them less susceptible to fibrosis-driven mechanisms (42, 43). These findings are consistent with preclinical studies demonstrating variability in plaque composition, microscopic structure, and susceptibility across vascular territories (42–44).

Understanding the factors that influence atherosclerosis at different anatomical sites is clinically important, as these differences directly impact the rate and nature of clinical events. For instance, the localization of SAD plays a pivotal role, with carotid arteries being more prone to foam cell lesions and lipid-rich plaques than femoral arteries. Accordingly, lipid-rich plaques are significantly more common in the carotid territory, particularly among patients who die from coronary atherosclerosis (42). Non-invasive magnetic resonance imaging studies have also demonstrated notable differences in plaque composition between vascular territories, revealing that carotid plaques tend to exhibit larger necrotic cores and more frequent hemorrhagic areas compared to those in the femoral arteries (43). Therefore, our findings align with prior research, such as the PDAY (Pathological Determination of Atherosclerosis in Youth) study, which highlighted that cardiovascular risk factors exert variable effects across different vascular sites (44).

Our findings also add to the growing evidence suggesting variability in the predictive power of liver fibrosis indices. FIB-4 index emerged as a strong independent predictor of carotid plaque presence and burden, while the NFS was also independently associated with carotid atheromatosis, albeit with somewhat weaker effect estimates. This discrepancy likely arises from differences in the indices’ sensitivity to advanced fibrosis versus metabolic factors, such as BMI and diabetes, which are integral to the NFS formula (19–21). Beyond liver- and site-specific mechanisms, our findings also support the interpretation of FIB-4 and NFS as integrative markers of cumulative, age-related cardiometabolic and fibrotic burden. In our cohort, the intermediate–high FIB-4 category was significantly older and exhibited a more adverse cardiometabolic profile. This observation is consistent with histology-based data in MASLD, where the prevalence and severity of fibrosis increase in a stepwise fashion with the number of metabolic comorbidities, and the count of these comorbidities independently predicts significant fibrosis (45). Similarly, hypertension [1.92 (1.17 to 3.16), p <0.001] and diabetes mellitus [2.00 (1.22 to 3.28), p <0.001] significantly contributed to advanced fibrosis (≥ F3) on multivariate analysis among 458 patients diagnosed with MASLD via liver biopsy (46). Taken together, these observations reinforce the utility of fibrosis scores, particularly FIB-4 and NFS, as reliable, non-invasive markers for identifying individuals at risk of subclinical carotid atherosclerosis, even in populations without overt liver or cardiovascular disease.

Atherosclerosis is a multifaceted process influenced by genetic, metabolic, and environmental factors. The interplay between systemic inflammation, oxidative stress, and metabolic dysfunction likely drives the progression of carotid atheromatosis. Emerging genetic evidence supports this notion in MASLD, showing that variants like PNPLA3 and TM6SF2, while strongly associated with hepatic fat and fibrosis, do not independently increase CVD risk in the absence of metabolic syndrome (47–49). This suggests that MASLD’s cardiovascular impact is predominantly mediated by systemic metabolic and fibrotic abnormalities rather than direct hepatic effects. Gender-specific differences also warrant attention. In our study, liver fibrosis (as estimated by the FIB-4 index) was independently associated with carotid plaque burden in men but not in women. This finding is consistent with the recognized sexual dimorphism in cardiovascular risk, whereby premenopausal women are relatively protected from atherosclerosis due to estrogen’s vasoprotective and anti-inflammatory effects. However, postmenopausal hormonal changes, together with an increase in visceral fat distribution and loss of muscle mass, tend to align women’s cardiovascular risk with that of men over time (50–52). The absence of a significant association in women in our study may reflect both a lower overall fibrosis burden and the complex interplay between hormonal status, metabolic factors, and vascular remodeling. Interestingly, in women, prediabetes and the presence of obesity remained significant independent predictors of carotid plaque, all of which are closely related to overall and central adiposity. Future studies including hormonal data or menopausal status could help clarify these sex-specific differences. Taken together, these findings underscore the need for sex-specific approaches when using liver fibrosis indices for cardiovascular risk stratification.

**Figure 2:**
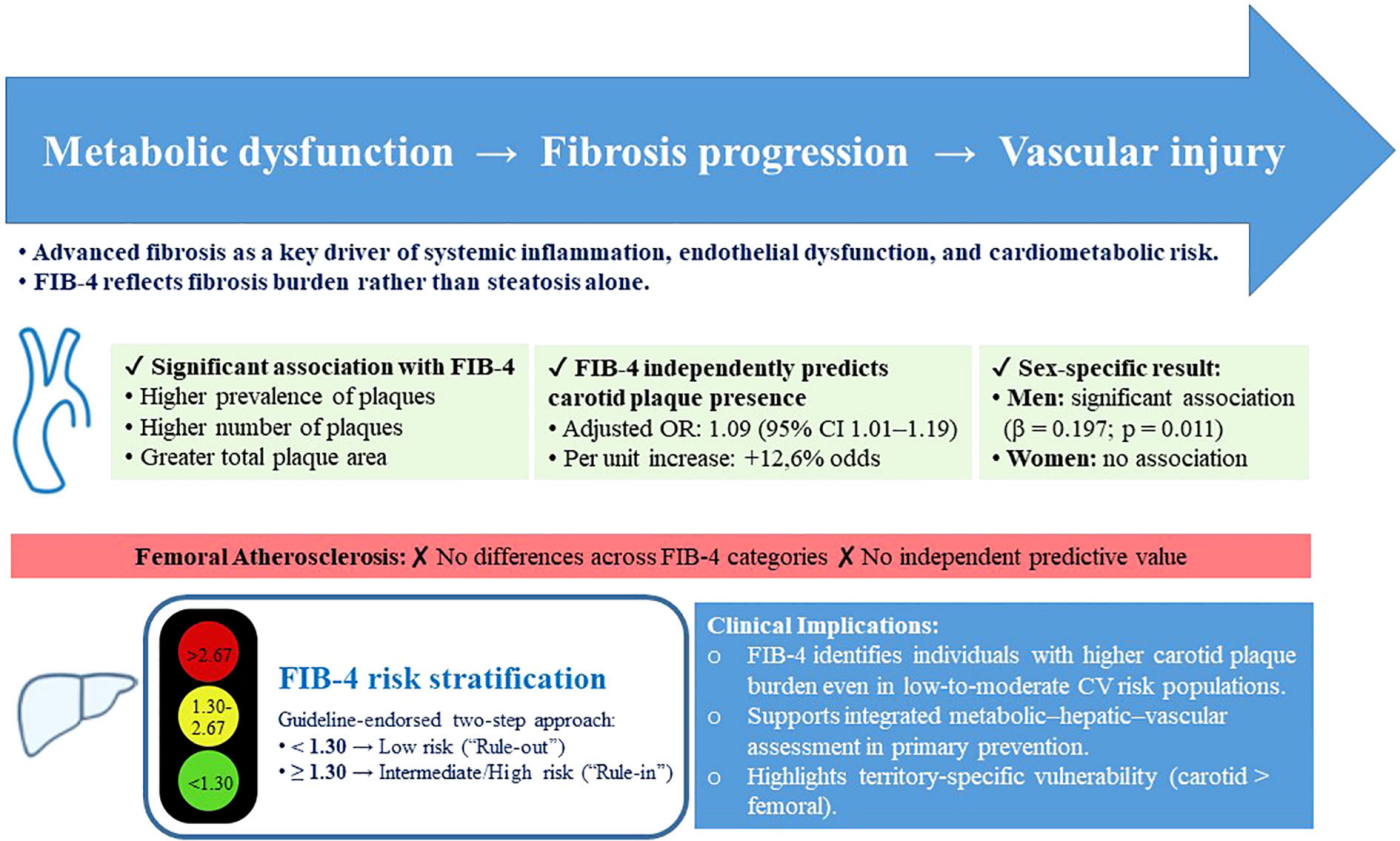
Central illustration: Association between FIB-4 and vascular atherosclerotic burden across carotid and femoral territories.

Our study has several limitations. First, its cross-sectional design does not allow for causal inference. Second, liver fibrosis was assessed using non-invasive indices rather than biopsy, which remains the diagnostic gold standard. Other more accurate non-invasive alternatives, such as transient elastography or the Enhanced Liver Fibrosis (ELF) score were also not used. Third, potential confounding by age should be acknowledged, given that age is a component of FIB-4 formula. Fourth, the number of participants with a high probability of advanced liver fibrosis based on FIB-4 scores was relatively small, which precluded treating this group as a separate analytical category. Fifth, atherosclerosis evaluation was confined to carotid and femoral territories, excluding other regions such as coronary arteries. Finally, potential survivor or selection bias cannot be entirely ruled out. Nonetheless, the study’s strength lies in its focus on a large, well-characterized cohort of individuals at low-to-moderate cardiovascular risk, without previous liver disease, providing valuable insights into the relationship between liver fibrosis and vascular health in the primary prevention setting. A central illustration summarizing the conceptual framework and main findings of the study is provided in **Figure 2**.

In summary, this study highlights the site-specific relationship between liver fibrosis and atherosclerosis, with advanced fibrosis preferentially associated with carotid plaque formation. These findings underscore the utility of non-invasive indices of liver fibrosis, particularly FIB-4, as a potential tool for identifying individuals at higher risk of subclinical atherosclerosis, even in populations without known liver disease. Future research should prioritize longitudinal studies to elucidate the temporal and causal links between liver fibrosis and vascular disease progression. Additionally, exploring the underlying mechanisms driving site-specific atherogenesis will be crucial for developing tailored therapeutic interventions.

## Data Availability

The raw data supporting the conclusions of this article will be made available by the authors, without undue reservation.

## Group member of ILERVAS project collaborators

Eva Miquel, Marta Ortega, Ferran Barbé, Jordi de Batlle, Silvia Barril, Manuel Portero-Otín, Mariona Jové, Josep Franch-Nadal, Esmeralda Castelblanco, Pere Godoy, Montse Martinez- Alonso, and Cristina Farràs.
