# Supplementary material for: Association of estimated liver fibrosis with carotid but not femoral atherosclerotic burden: the ILERVAS cohort
Source: Front Endocrinol (Lausanne). 2026 Jan 6;16:1651689. doi: 10.3389/fendo.2025.1651689 (PMC12816284; doi:10.3389/fendo.2025.1651689)
Supplement: Supplementary file 1 [file DataSheet1.pdf]

Supplemental Table 1. Lineal regression model for the analysis between number of carotid plaques and FIB4-index in men.

	Odds Ratio (95% confidence interval)	p
**Pulse pressure (mm Hg)**	1.04 (1.03 to 1.36)	<0.001
**Prediabetes (yes/no)**	1.23 (0.98 to 1.54)	0.069
**LDL-cholesterol (mg/dl)**	1.006 (1.002 to 1.009)	<0.001
**Triglycerides (mg/dl)**	1.001 (0.99 to 1.002)	0.145
**Obesity (yes/no)**	1.12 (0.90 to 1.39)	0.323
**Smoking**	1.35 (1.10 to 1.66)	0.005
**FIB-4 index**	1.18 (1.03 to 1.36)	0.018
**Test of fit Hosmer-Lemeshow**		0.1566
**Area under the ROC curve**		0.6271

LDL: low density lipoprotein.

Supplemental Table 2. Lineal regression model for the analysis between number of carotid plaques and FIB4-index in women.

	Odds Ratio (95% confidence interval)	p
**Pulse pressure (mm Hg)**	1.03 (1.02 to 1.04)	<0.001
**Prediabetes (yes/no)**	1.32 (1.09 to 1.60)	0.005
**LDL-cholesterol (mg/dl)**	1.004 (1.001 to 1.007)	0.006
**Triglycerides (mg/dl)**	1.0001 (0.99 to 1.002)	0.804
**Obesity (yes/no)**	1.25 (1.05 to 1.49)	0.007
**Smoking**	1.32 (1.07 to 1.62)	0.010
**FIB-4 index**	1.11 (0.99 to 1.24)	0.067
**Test of fit Hosmer-Lemeshow**		0.2663
**Area under the ROC curve**		0.6235

LDL low density lipoprotein.
